# Monozygotic twins with neuroblastoma MS have a similar molecular profile: a case of twin-to-twin metastasis

**DOI:** 10.1038/s41416-019-0594-3

**Published:** 2019-10-11

**Authors:** Margaret Shatara, Ana C. Xavier, Alan Dombkowski, Daniela Cukovic, Janet M. Poulik, Deniz Altinok, Yubin Ge, Jeffrey W. Taub

**Affiliations:** 1 Carman and Ann Adams Department of Pediatrics, Children’s Hospital of Michigan/Wayne State University School of Medicine, Detroit, MI USA; 2 Division of Hematology/Oncology, Department of Pediatrics, University of Alabama at Birmingham, Birmingham, AL USA; 3 Department of Oncology, Wayne State University School of Medicine, Detroit, MI USA

**Keywords:** Cancer genomics, Oncogenes

## Abstract

Fetoplacental neuroblastoma metastasis has been postulated as a mechanism accounting for concordant cases where one twin develops a primary tumour and the second twin manifests the disease without an identifiable primary site. These tumours may originate and spread concomitantly due to the same genetic background shared by monozygotic twins. This study investigated the molecular profile of stage MS neuroblastoma presenting concomitantly in monozygotic twins. Comparative genomic hybridisation (aCGH) was done for each of the twin liver tumour and peripheral blood samples at diagnosis. Comparison of copy-number variation (CNV) regions revealed a set of CNVs that were common to both tumour specimens and not apparent in the blood. The CNV signature in both twins’ tumours was highly similar, suggesting a common clonal origin. Additional findings included large deletion of chromosome 10 and amplification of chromosome 17. Notably, both liver samples had amplification of a short region involving *DEIN* (chromosome 4q34.1). Similar CNVs strongly support a common clonal origin and metastatic spread from one twin to the other. *DEIN* is a long-coding RNA (IncRNA) that has been found highly expressed in stage MS neuroblastoma and is likely involved in biological processes such as cell migration and metastasis.

## Background

Neuroblastoma (NBL) is the most common extracranial solid tumour of childhood. Concordant or discordant occurrence of NBL in monozygotic twins has been described in the literature.^1,2^ Fetoplacental metastasis has been postulated as a mechanism to account for concordant cases where one twin develops a primary adrenal tumour and the second twin manifests the disease without an identifiable primary site.^1,2^ However, those tumours may originate and arise concomitantly due to the same genetic background shared by monozygotic twins, suggesting a predisposition to develop NBL. We present a case of stage MS NBL presenting concomitantly in monozygotic monochorionic twins.

Twin B was a 2-month-old who presented with one-week history of abdominal distention and decreased oral intake. Twin A presented with similar complaints one day after Twin B. Both twins were diagnosed with stage MS NBL (MYCN non-amplified, favourable Shimada histology; Twin B with adrenal, liver and bone marrow disease; Twin A with liver and bone marrow disease) and were treated with cycles of carboplatin, etoposide alternating with carboplatin, cyclophosphamide and doxorubicin.^3^ Following two cycles of chemotherapy, interim-staging scans revealed the interval decrease in right adrenal (Twin B) and liver (Twins A and B) masses, declining urine vanillylmandelic acid/homovanillic acid (uVMA/HVA) and minimal (Twin B) or no (Twin A) bone marrow tumor infiltration. Following four cycles of chemotherapy, both twins had normalisation of uVMA/HVA and complete or near-complete NBL image resolution. Both twins are now healthy long-term survivors.

## Methods

Array comparative genomic hybridisation (aCGH) for each of the twin liver tumour and peripheral blood (PB) samples was performed as per the manufacturer’s protocol.^4^ Microarray images were analysed by using linear and lowess normalisation to provide log ratio values for each probe, with the Promega standard used as the reference channel. aCGH data were imported into Genomic Workbench software (Agilent Technologies^©^) for identification of structural variants. Regions of copy-number variation (CNV) were detected in each sample by using the ADM-1 algorithm, threshold = 5.0, and human genome build hg19. Normalised log ratios of probes in CNV regions were then exported to CIRCA software (omgenomics.com) for visual comparison between samples.

## Results

**Fig. 1:**
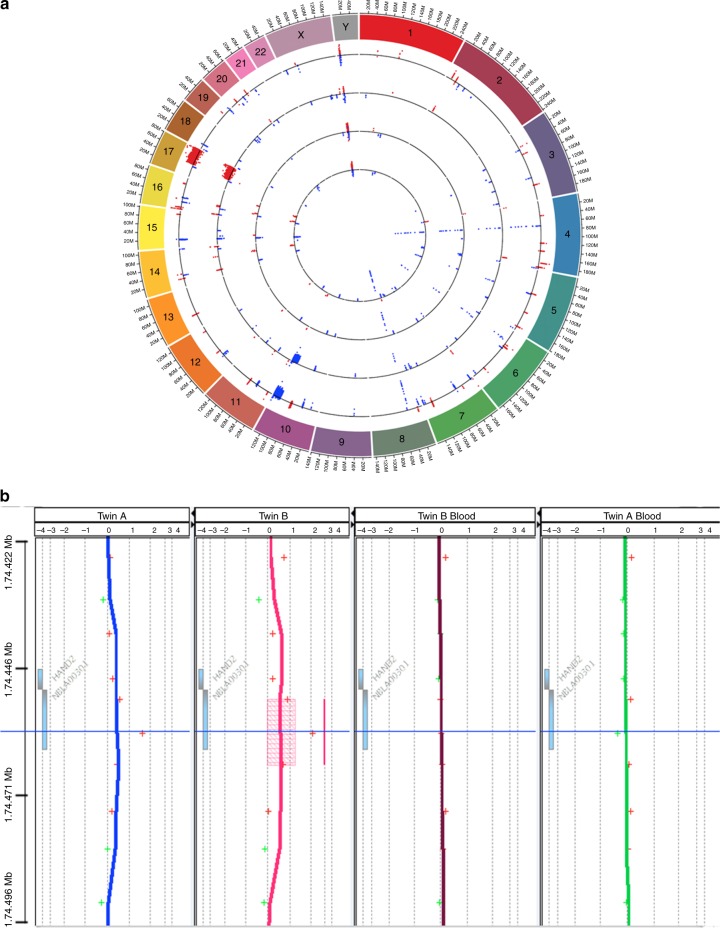
**a** aCGH data. On each ring, the data points are shown for regions of CNV. Red points indicate an amplification. Blue points represent deletions. The CNV calls were made with respect to a commercial reference standard Promega that is comprised of DNA from numerous non-disease female individuals. From outer to inner: Twin A liver tumour sample, Twin B liver tumour sample, Twin A PB sample and Twin B PB sample. **b** A short region of amplification on gene locus NBLA00301, representing the *DEIN* gene (*HAND2-AS1*, localised to 4q34.1, MIM617240). This region was flagged in only Twin B by the ADM-1 algorithm, despite the clear amplification apparent in both twin tumour samples. CNV, copy-number variant; PB, peripheral blood; aCGH, array comparative genomic hybridisation

Comparison of CNV regions revealed a set of CNVs that were common to both tumour specimens and not apparent in the blood (Fig. 1a), indicating somatic variants. The CNV signature in both twin tumours was highly similar. Prominent among the CNV regions were a large deletion of chromosome 10 and large amplification of chromosome 17, commonly found in NBL cases.^5^ Conventional CNV detection algorithms are susceptible to noise in aGCH data and have a high rate of false negatives and positives, particularly for short CNVs. To identify high-confidence short CNVs, we applied an algorithm that was developed *in-house* to the aCGH data. A notable finding was a short region of amplification on locus NBLA00301, representing the *DEIN* gene (*HAND2-AS1*, localised to 4q34.1, MIM617240) (Fig. 1b).

## Interpretation

Stage MS NBL is usually a sporadic disease with distinct clinical and molecular features that are different from neuroblastoma stage IV.^6,7^ The occurrence of synchronous tumour in monozygotic twins may suggest the presence of a common genetic background as a predisposition factor for NBL genesis. Furthermore, fetoplacental metastasis is considered when monozygotic monochorionic twins have similar tumour pathology, but only one with identifiable primary tumour, presenting spontaneously, or shortly after.^1,2^ A similar mechanism was postulated for concordance of leukaemia in twins.^8,9^ Several molecular markers are considered evidence for common clonality and common origin of tumourigenesis.^9^ Nearly identical CGH and CNV profiles provide substantial evidence of the clonal origin of the malignant cells.^10–12^

Previous aCGH analysis of NBL in a set of monozygotic twins revealed few differences between the primary adrenal versus liver tumours, supporting metastatic spread.^2^ In our report, similar CNVs between both twin tumour samples also strongly support a common clonal origin.

Interestingly, both twin liver tumours had a 4q34.1 amplification, involving *DEIN*. *DEIN* is a long noncoding RNA (IncRNA) tightly linked to *HAND2*, a gene that functions as a key regulator of neurogenesis in the developing sympathetic nervous system.^13^ *DEIN* has also be found to be a candidate IncRNA related to metastasis in hepatocellular carcinoma.^14^ Stage MS NBL is an entity defined by its metastatic pattern confined to the skin, liver and bone marrow, in addition to the tumour unique capability to undergo spontaneous regression in young children.^15,16^ High expression of *DEIN* variants has been found to occur exclusively in stage MS NBL, potentially implicating this gene and its variants in biological processes such as cell migration and metastasis in stage MS NBL.^13^

Of note, no abnormalities involving genes previously described in familial cases of NBL, such as paired-like homeobox 2B (*PHOX2B*), and the anaplastic lymphoma kinase (*ALK*) were identified here, also supporting its sporadic nature.^17^ It is interesting that for the twins, tumour preferentially metastasised to similar organs (e.g. liver, bone marrow), which is the pattern for stage MS NBL. Additional studies are needed to clarify the role of IncRNA in metastatic spread of stage MS NBL.

## Method

Comparative genomic hybridisation (CGH) was performed for each of the twin tumour specimens and their respective peripheral blood samples. Agilent Human Genome aCGH 1 × 244k (p/n G4411B) and Agilent Genomic DNA Enzymatic labeling Kit (p/n 5190-0453) were used in the direct method of the oligo aCGH workflow as described in Agilent protocol “Oligonucleotide Array-Based CGH for Genomic DNA Analysis”.^4^ Direct labelling of 1.5 µg of gDNA started with fragmentation with AluI and RsaI enzymes, followed by fluorescent labelling of fragmented gDNA with Cyanine 3-dUTP or Cyanine 5-dUTP and Exo-Klenow fragment, and cleanup of labelled genomic DNA. Genomic DNA samples from tumours and peripheral blood were labelled with Cy5, while standard female gDNA from Promega was labelled with Cy3. Hybridisation mixture contained Cy5- and Cy3-labelled gDNA in 158 µl, 50 µl of human Cot-1 DNA, 50 µl of Agilent 10× blocking agent and 250 µl of Agilent 2× hybridisation buffer. After incubation at 95 °C for 3 min, the hybridisation mixture was incubated at 37 °C for 30 min, and then immediately added to an array in an Agilent SureHyb hybridisation chamber to co-hybridise on the array for 40 h at 65 °C and 20 rpm. The arrays were washed with Agilent Oligo aCGH Wash buffer 1 and 2 according to the Agilent protocol and scanned at 5-µm resolution with an extended dynamic range by using an Agilent dual-laser scanner. Microarray images were analysed with Agilent Feature Extraction software v10.7.1.1 by using linear and lowest normalisation to provide log ratio values for each probe, with the Promega standard used as the reference channel. Array CGH data were imported into Genomic Workbench software (Agilent Technologies) for identification of structural variants. Regions of CNV were detected in each sample by using the ADM-1 algorithm, threshold = 5.0, and human genome build hg19. Normalised log ratios of probes in CNV regions were then exported to CIRCA software (omgenomics.com) for visual comparison between samples.

## Data Availability

Data supporting the results are presented at the end of this paper and in Fig. 1. Detailed method is presented at the end of this paper.
